# Alarming Symptoms Following the Administration of One Twenty-Fourth of a Grain of Merck’s Hyoscyamine

**Published:** 1891-10

**Authors:** Hugh Hagan

**Affiliations:** 151 Peachtree street, Atlanta, Ga.


### ALARMING SYMPTOMS FOLLOWING THE ADMINISTRATION OF ONE TWENTY-FOURTH OF A GRAIN OF MERCK'S HYOSCYAMINE.

BY HUGH HAGAN, M. D.

---

E. C. M., aged 57 years, male and married, consulted me about four weeks since in regard to his nervous condition.

He presented a typical case of paralysis agitans. I prescribed bi-weekly suspension, recommended and practiced by Professor Benedict, of Vienna, tri-weekly applications to the spinal column of a constant galvanic current of 15 cells, about 10 milliamperes, and about 20 drops of an aqueous solution of hyoscyamine, 1 grain to the ounce, thus administering 1-24 grain.

The afternoon of the day I prescribed, the wife of the patient came in great haste and excitement to my office, saying, "her husband was dying, and that the medicine I had prescribed had poisoned him." I hastened to the house, and found the patient much prostrated, face flushed, complaining of severe headache, soreness of throat and tongue, total blindness , dizziness, tinnitus aurium and great muscular weakness. The heart was beating 85, and regular. The respiration was unaffected, save for a slight increase in frequency, and there was considerable mental confusion. I immediately administered one-quarter of a grain of the sulphate of morphia subcutaneously, and in the course of three hours one ounce whisky. Very soon after this the symptoms began to abate, and near midnight I left him quite comfortable. He afterwards stated "that about one hour after taking the 20 drops as prescribed, he became dizzy and could not see." I had previouslv warned him of the possible effects of the drug, thus saving him the apprehension he would otherwise have suffered. I afterwards learned he possessed an idiosyncrasy for quinine, having frequently suffered severe headache, pruritus, urticaria and erythenia after taking 5 grains of this drug. The patient is now doing well under suspension, electricity and a pill of half grain of zinci phosphuri.

By way of apology for citing this case, I deemed it of sufficient interest, as such authorities as Lawson, Oulemont and Laurent advise much larger doses, and Lawson has given as high as 1 1-2 grains in cases of cerebral excitement, and though we all know in cases of mania, and the like, a marked increase in toleration is present, yet one would hardly expect such marked symptoms from so modorate a dose, and that given by the mouth, I have myself often administered 1-10 grain, and save this time have never seen any untoward symptoms.

*151 Peachtree street, Atlanta, Ga.*

